# S159. REDUCED PROCESSING SPEED IN SCHIZOPHRENIA IS MEDIATED BY WHITE MATTER INTEGRITY

**DOI:** 10.1093/schbul/sby018.946

**Published:** 2018-04-01

**Authors:** Saetbyeol Cha, Woon Yoon, Seung-Hyun Shon, Jungsun Lee

**Affiliations:** Asan Medical Center

## Abstract

**Background:**

Meta-analysis suggest that processing speed deficit is the largest single cognitive impairment in schizophrenia. Processing speed predicts functional outcome and indicates a vulnerability marker for schizophrenia. Several authors have proposed that abnormalities in white matter is related to reduced processing speed in schizophrenia. The purpose of this research was to investigate the relationship between processing speed and structural properties of white matter pathways in schizophrenia and healthy controls.

**Methods:**

The data using this study were from the SchizConnect. Participants included 64 patients with schizophrenia and 71 healthy controls. Diffusion tensor imaging(DTI) method was used to measure fractional anisotropy along white matter tracts. Group differences in white matter integrity-inferred from fractional anisotropy (FA), processing speed, verbal memory were examined. Mediation analysis were applied to inspect the relationship between FA and cognitive performance.

**Results:**

Participants with schizophrenia had significantly reduced processing speed, verbal memory deficits, and whole-brain fractional anisotropy deficit. There were significant group differences in white matter integrity of the left thalamus occipital, right extreme capsule, and right thalamus occipital. FA in left thalamus occipital and right extreme capsule mediated group differences in processing speed, but not other cognitive domains.

**Discussion:**

Study findings indicate that mediation effect of processing speed is regional tract-specific. These finding suggest that the structural integrity of white matter tracts associated with left thalamus occipital, right extreme capsule is closely related to reduced processing speed in schizophrenia, but not verbal memory and verbal learning.

